# Inhibition of Beta-Amyloid Fibrillation by Luminescent Iridium(III) Complex Probes

**DOI:** 10.1038/srep14619

**Published:** 2015-09-30

**Authors:** Lihua Lu, Hai-Jing Zhong, Modi Wang, See-Lok Ho, Hung-Wing Li, Chung-Hang Leung, Dik-Lung Ma

**Affiliations:** 1 Department of Chemistry, Hong Kong Baptist University, Kowloon Tong, Hong Kong, China; 2 State Key Laboratory of Quality Research in Chinese Medicine, Institute of Chinese Medical Sciences, University of Macau, Macao, China

## Abstract

We report herein the application of kinetically inert luminescent iridium(III) complexes as dual inhibitors and probes of beta-amyloid fibrillogenesis. These iridium(III) complexes inhibited Aβ_1–40_ peptide aggregation *in vitro*, and protected against Aβ-induced cytotoxicity in neuronal cells. Furthermore, the complexes differentiated between the aggregated and unaggregated forms of Aβ_1–40_ peptide on the basis of their emission response.

Over the past few years, vast efforts have been dedicated to the development of imaging probes and inhibitors for the diagnosis and treatment of Alzheimer’s disease, which is a highly common neurodegenerative disorder characterized by the progressive loss of cognitive ability^1^,^2^. One of the strategies for the treatment of Alzheimer’s disease is the development of inhibitors that prevent the misfolding and self-assembling aggregation of monomeric Aβ peptides into neurotoxic fibrils^1^. Many inhibitors have been reported to inhibit Aβ peptide aggregation^3^,^4^,^5^,^6^, such as short peptides^7^,^8^, organic molecules^9^,^10^,^11^,^12^,^13^,^14^,^15^,^16^, supramolecular cucurbit[7]uril^17^, polyoxometalates^18^,^19^, nanoparticles^20^ and ligand-functionalized quantum dots^21^. Meanwhile, the development of new diagnostic probes for Aβ fibrillation has also been an important goal. The early diagnosis of Alzheimer’s disease may allow for palliative treatment to alleviate symptoms or to slow down the progression of the disease. A number of luminescent probes have been developed for the specific labeling and imaging of Aβ plaques, such as luminescent conjugated polythiophenes^22^ and common fluorescent dyes^23^,^24^,^25^,^26^,^27^,^28^,^29^,^30^,^31^. Dual-role imaging agents and aggregation inhibitors of Aβ may serve a bifunctional purpose for Alzheimer’s disease, as the inhibition of Aβ fibrillogenesis may be monitored without the need for an extraneous labeling agent. However, to our knowledge, only a few examples of dual-function inhibitors and probes of Aβ have been reported in recent years. Organic dyes commonly used for labeling Aβ fibrils, including Congo Red and Thioflavine T (ThT), have been shown to inhibit Aβ aggregation at high concentrations of dyes^32^.

Luminescent transition metal complexes have found emerging use for the design of chemical and biological sensors^33^,^34^ in view of their useful photophysical properties, such as tunable excitation and emission wavelengths (from blue to red), high luminescent quantum yields, and relatively long phosphorescent lifetimes^33^. At the same time, transition metal complexes have been increasingly regarded as a promising alternative to organic compounds as therapeutic agents for the treatment of human diseases^35^,^36^,^37^,^38^,^39^,^40^,^41^. Due to the well-defined three-dimensional structure of transition metal complexes, highly specific interactions between metal complexes and biomolecules can be obtained through modification of the steric and electronic nature of the organic ligands surrounding the metal centre. In the context of Alzheimer’s disease, metal complexes such as Pt(II)^42^,^43^,^44^,^45^,^46^,^47^,^48^, binuclear Ru(II)–Pt(II)^49^, Ru(II)^50^,^51^,^52^, Co(III)^53^ and Ir(III) or Rh(III) solvato^54^ complexes have been reported to inhibit the aggregation of Aβ peptide^55^,^56^,^57^. Furthermore, luminescent metal complexes such as Ru(II)^58^,^59^, Re(I)^60^ complexes have also been used to monitor the Aβ fibrillation process^30^,^55^. However, few transition metal complexes have been reported to be dual inhibitors and luminescent probes of Aβ. Moreover, while iridium(III) complexes have been widely used as probes for various biomolecules, their application as dual inhibitors and probes of beta-amyloid fibrillogenesis has not been previously described.

Our group has previously reported the application of Ir(III) and Rh(III) solvato complexes as inhibitors of amyloid fibrillogenesis and as luminescent probes for Aβ peptide^54^. Mass spectrometry experiments indicated that the Rh(III) solvato complex formed 1:1 covalent adducts with Aβ_1–40_, presumably through coordination of the N-donor histidine residue of Aβ_1–40_ to the Rh(III) center via displacement of the aqua ligands. Encouraged by our previous results with Group 9 solvato compounds, we sought to investigate the ability of kinetically inert Ir(III) complexes to function as dual imaging agents and inhibitors of Aβ peptide aggregation. We report herein the synthesis of a series of luminescent Ir(III) complexes containing various C^N and N^N ligands, and their ability to detect and inhibit Aβ fibrillation. We envisage that these kinetically inert Ir(III) complexes may be developed as a novel class of dual-purpose probes and inhibitors of Aβ aggregation for the effective diagnosis and/or treatment of Alzheimer’s disease.

## Results

**Figure 1:**
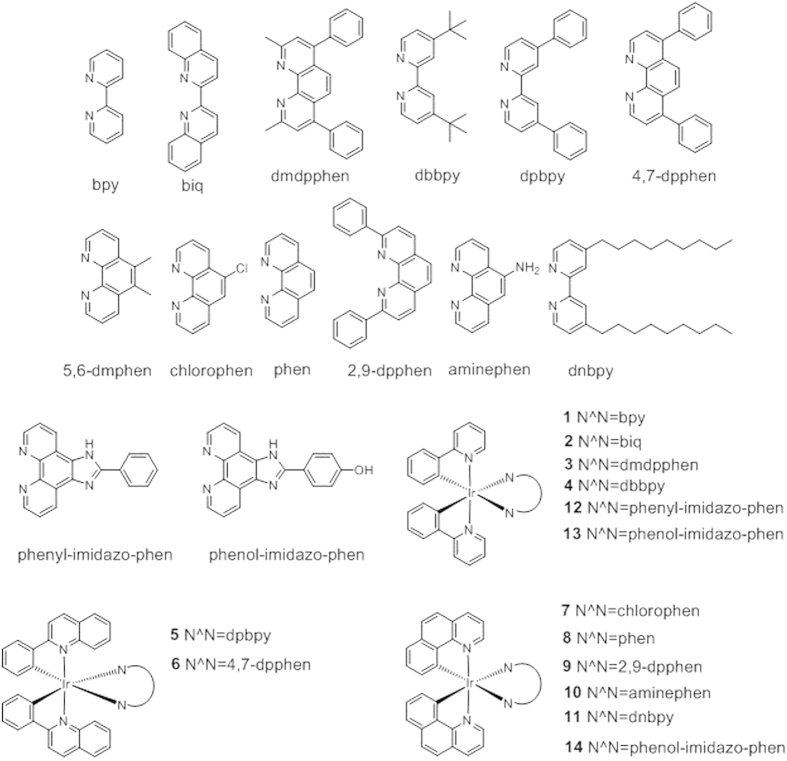
Chemical structures of the luminescent Ir(III) complexes 1–14 which were synthesized and evaluated in this study.

A library of twelve luminescent Ir(III) complexes (**1**–**12**, Fig. 1) were initially examined for their ability to interact with different forms of Aβ_1–40_ by emission titration. Of these twelve complexes, **12** bearing the 2-phenyl-1H-imidazo[4, 5-*f*][1,10]phenanthroline (phenyl-imidazo-phen) N^N ligand displayed the *I*_fibril_/*I*_monomer_ ratio (>1.5), indicating that it possessed the highest distinguishing ability for detecting Aβ_1–40_ fibrils over Aβ_1–40_ monomers (Figure S1). However, **1**–**11** were unable to effectively discriminate between Aβ_1–40_ monomers and fibrils. Based on the structure of **12**, we designed and synthesized **13** and **14**. The characterization and photophysical properties of **1**–**14** are given in the ESI (Table S1).

**Figure 2:**
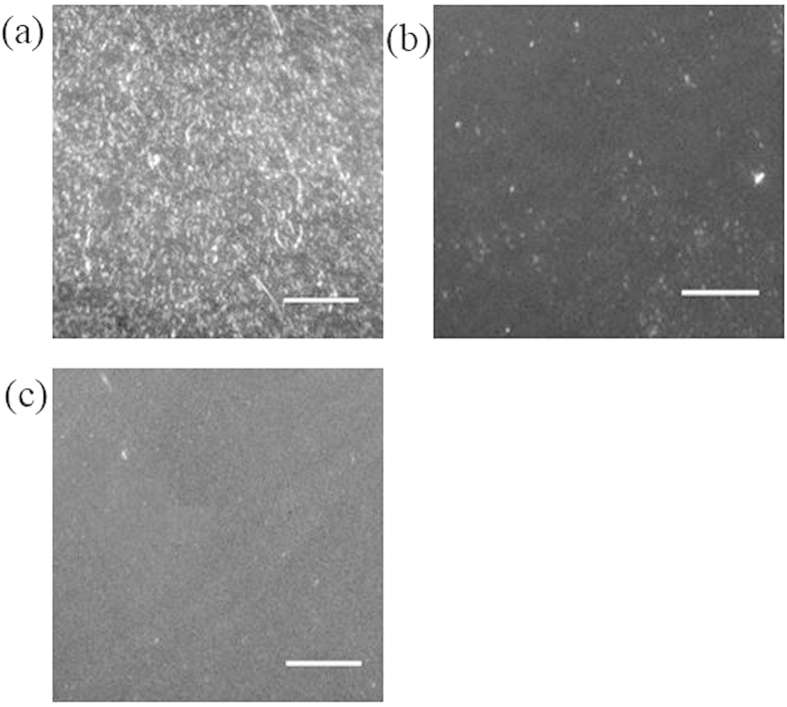
Inhibition of seed-mediated Aβ_1–40_ fibril growth by Ir(III) complexes. TIRFM images of Aβ_1–40_ fibrils grown in the (**a**) absence and presence of (**b**) **13**, (**c**) **14** after incubation at 37 °C for 1 h. The scale bar for TIRFM is 20 μm.

**Figure 3:**
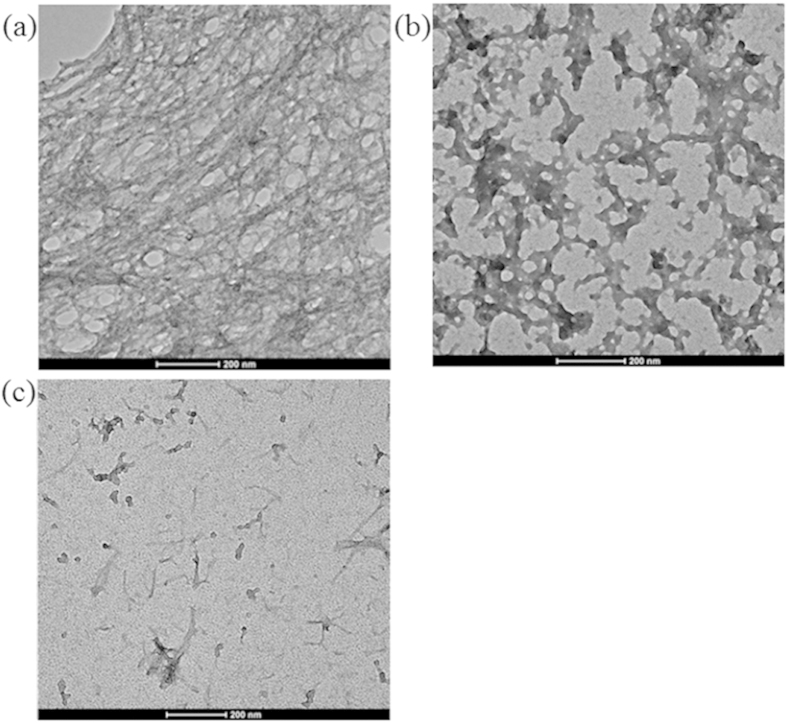
Inhibition of seed-mediated Aβ_1–40_ fibril growth by Ir(III) complexes. TEM images of Aβ_1–40_ fibrils grown in the (**a**) absence and presence of (**b**) **13**, (**c**) **14** after incubation at 37 °C for 1 h. The scale bar for TEM is 200 nm.

Complexes **12**–**14** were investigated for their ability to inhibit the fibrillogenesis of Aβ_1–40_. Given that these complexes could detect Aβ_1–40_ fibrils over Aβ_1–40_ monomers, the kinetics of Aβ_1–40_ aggregation in the presence of the complexes could be monitored without the need for an external labeling agent. The results revealed typical sigmoidal growth curves in seed-mediated Aβ fibrillation when Aβ_1–40_ monomers was incubated with ThT or **12**–**14** (Figure S2a and S2b). However, fibrillogenesis was partial retarded by **13** and completely inhibited by **14**, indicating that these two complexes were able to inhibit Aβ_1–40_ peptide aggregation. As both **13** and **14** contain the phenol-imidazo-phen N^N ligand, the effect of that ligand alone on Aβ_1–40_ peptide aggregation was investigated. The results showed that the presence of the N^N ligand alone had no inhibitory effect on Aβ_1–40_ peptide aggregation, highlighting the importance of the iridium(III) metal center in maintaining the octahedral structure of the active complexes (Figure S2c). Moreover, total internal reflection fluorescence microscopy (TIRFM) with laser excitation was used to observe the ThT-labelled fibrils with high sensitivity. In the absence of Ir(III) complexes, the fluorescence images of the Aβ_1–40_ peptides incubated by the seed-mediated method showed dense spots, corresponding to newly genereted Aβ_1–40_ fibrils (Fig. 2a). In contrast, these spots partially or completely disappeared when the samples were treated with **13** (Fig. 2b) or **14** (Fig. 2c), indicating that **13** and **14** could partially or completely inhibit the fibrillogenesis of amyloid peptides, respectively. Transmission electron microscopy (TEM) images further confirmed the ability of the Ir(III) complexes to inhibit seed-mediated Aβ aggregation. In the control experiment (Fig. 3a), Aβ_1–40_ grewinto thick, dense and long hair-like fibrils. However, when the sample was incubated with **13,** the TEM image showed short flake**-**like fibris (Fig. 3b), indicating that **13** was able to partially inhibit the Aβ_1–40_ aggregation. Furthermore, incubation of the sample with **14** resulted in the complete absence of elongated fibrils, and only the preformed fibril seeds were observed (Fig. 3c), indicating the complete inhibition amyloid fibrillogenesis by **14**. Moreover, the fibrillogenesis kinetics of Aβ_1–40_ in the presence of various concentrations of **14** showed that the inhibition of Aβ_1–40_ aggregation by **14** was concentration-dependent (Figure S3). At 50 μM of **14**, the inhibition of 25 μM of Aβ_1–40_ peptides was nearly completely inhibited.

**Figure 4:**
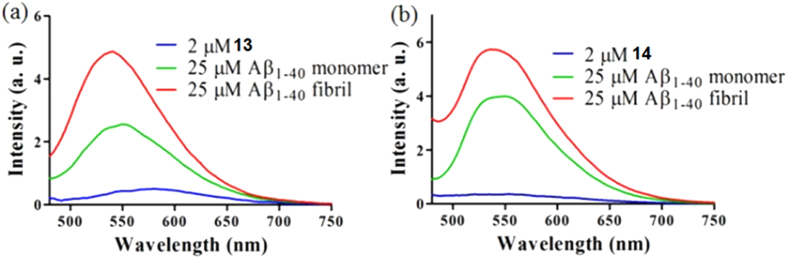
Luminescence response of 2 μM of (a) **13** and (b) **14** in the absence or presence of 25 μM Aβ_1–40_ monomers or fibrils. λ_Ex_ = 360 nm.

Encouraged by the promising activity of **13** and **14** against Aβ aggregation, we investigated that the luminescence behaviour of **13** and **14** towards different forms of Aβ_1–40_. The results showed that both complexes displayed a significantly enhanced luminescence response in the presence of the Aβ_1–40_ monomers or fibrils (Fig. 4). **13** exhibited *ca.* 6- and 12-fold emission enhancements at λ_max_ = 540 nm in the presence of comparable mass concentrations of Aβ_1–40_ monomers and fibrils, respectively. On the other hand, **14** showed *ca.* 11- and 18-fold emission enhancements at λ_max_ = 540 nm in the presence of Aβ_1–40_ monomers and fibrils. Taken together, these two Ir(III) complexes showed luminescence enhancement to the presence of Aβ_1–40_ monomers and fibrils to different extents. We presume that this behaviour of the Ir(III) complexes towards the Aβ_1–40_ peptide may be due to the ability of the complexes to bind to a hydrophobic region within the peptide, thus protecting the complexes from non-radiative decay by solvent quenching and thereby giving rise to an enhanced luminescence response. The differential luminescence response of the complexes towards Aβ_1–40_ monomers and fibrils may be due to the different microenvironments experienced by the Ir(III) complexes upon binding to the Aβ_1–40_ monomers or the fibrils.

ESI-TOF mass spectrometry experiments were performed to examine the binding of the Ir(III) complexes to Aβ_1–40_ peptide. The mass spectrum of the Aβ_1–40_ monomer in the absence of the Ir(III) complexes shows two characteristic peaks at 1083 and 1444, corresponding to the 4+ and 3+ ionization states of the Aβ_1–40_ monomer, respectively (Figure S4a). However, incubation of the Aβ_1–40_ peptide with **13** (Figure S4b) or **14** (Figure S4c) produced no new peaks in the mass spectra besides those corresponding to the free complex (813 for **13** and 861 for **14**), suggesting that the Ir(III) complexes were not covalently bound to the Aβ_1–40_ peptide.

The cytotoxicity of the most potent Ir(III) complex **14** was examined using the 3-(4,5-dimethylthiazol-2-yl)-2,5-diphenyltetrazolium bromide (MTT) assay (Figure S5). Neuroblastoma cells (SH-SY5Y) were incubated in the presence of different concentrations for 24 h and cell viability was examined using the MTT assay. The IC_50_ value of **14** was estimated to be >100 μM at 24 h of exposure. Notably, these IC_50_ values are significantly higher than the concentration of **14** required for complete inhibition of Aβ_1–40_ peptide aggregation, suggesting the presence of a therapeutic window whereby Aβ_1–40_ peptide aggregation can be controlled without significant damage to brain cells.

**Figure 5:**
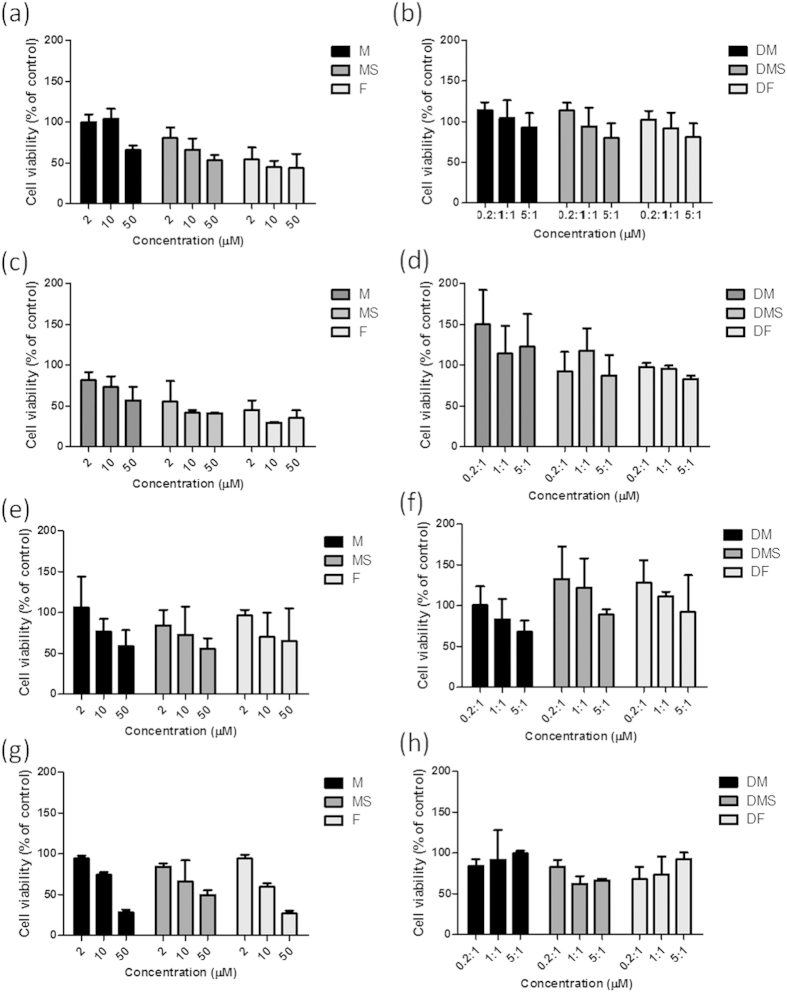
Neuroprotective effect of 14 against Aβ_1–40_ peptide-induced cytotoxicity towards (a–d) human neuroblastoma SH-SY5Y cells and (e–h) mouse primary cortical cells. Cell viability is expressed as a percentage of control cells exposed to 0.5% DMSO. The histograms show the cell viability of various concentrations of Aβ_1–40_ peptide monomer (M), Aβ_1–40_ peptide with seeded fibril (MS), and fibrillar Aβ_1–40_ peptide (F), in the presence of **14**. Various forms of Aβ_1–40_ peptide were incubated for (**a**,**b**,**e**,**f**) 2 h, and for (**c**,**d**,**g**,**h**) 24 h at [Aβ_1–40_]:[**14**] ratios of 0.2:1, 1:1, and 5:1.

The effect of **14** on Aβ_1–40_-induced cytotoxicity in SH-SY5Y cells and mouse primary cortical cells was also investigated. The cytotoxicity of three different forms of Aβ_1–40_ peptide in the presence and the absence of **14** were examined: Aβ_1–40_ peptide monomer (M), Aβ_1–40_ peptide monomer with seeded fibrils (MS) and Aβ_1–40_ fibril (F) (Fig. 5). The results showed that treatment of cells with different forms of Aβ_1–40_ peptides caused toxicity to SH-SY5Y cells and mouse primary cortical cells (Fig. 4a,c,e,g). Encouragingly, **14** exhibited a neuroprotective effect against the cytotoxicity induced by all three forms of Aβ_1–40_ peptide at [Aβ_1–40_]/[14] ratios of 0.2, 1.0, or 5.0 for SH-SY5Y cells (Fig. 5a,b) or mouse primary cortical cells (Fig. 5e,f) after 2 h of incubation. The neuroprotective effects of **14** were still observable after 24 h of incubation of **14** (Fig. 5c,d,g,h). As a negative control, we also investigated the effect of **12**, which showed no effect against amyloid aggregation, on Aβ_1–40_-induced toxicity. The results showed that **12** had no neuroprotective effect against cytotoxicity induced by all three forms of Aβ_1–40_ peptide at [Aβ_1–40_]/[12] ratios of 0.2, 1.0, or 5.0 in SH-SY5Y cells (Figure S6). Taken together, these data indicate that **14** displays neuroprotective effects against Aβ-mediated cytotoxicity when administered at a low enough dosage in SH-SY5Y cells and mouse primary cortical cells.

## Discussion

In conclusion, a library of 12 luminescent Ir(III) complexes containing various C^N and N^N ligands were initially screened as luminescent probes for Aβ_1–40_ peptide. Based on the ability of **12** for distinguishing Aβ_1–40_ fibrils over monomers, **13** and **14** were further synthesized and tested. The novel Ir(III) complex **14** emerged as the most potent candidate and was shown to inhibit Aβ_1–40_ peptide aggregation as revealed by a luminescence assay, as well as TIRFM and TEM imaging. Notably, Aβ_1–40_ peptide aggregation was nearly completely inhibited at 50 μM of **14**. A neuroprotective effect of **14** against Aβ_1–40_-induced cytotoxicity in SH-SY5Y cells and mouse primary cortical cells was also demonstrated. Using ESI-TOF mass spectrometry, we also showed **14** was not covalently bound to the Aβ_1–40_ peptide. Non-covalent probes may have a better safety index, lower cross-reactivity, and lower immunogenicity compared to covalently-binding molecules^61^,^62^,^63^. We envision that this work would open up new avenues for the development of dual-role imaging agents and aggregation inhibitors of Aβ for the treatment of Alzheimer’s disease.

## Methods

### Chemicals and materials

Reagents, unless specified, were purchased from Sigma Aldrich (St. Louis, MO) and used as received. Iridium chloride hydrate (IrCl_3_·xH_2_O) was purchased from Precious Metals Online (Australia).

### General experiment

Mass spectrometry was performed at the Mass Spectroscopy Unit at the Department of Chemistry, Hong Kong Baptist University, Hong Kong (China). Deuterated solvents for NMR purposes were obtained from Armar and used as received.

^1^H and ^13^C NMR were recorded on a Bruker Avance 400 spectrometer operating at 400 MHz (^1^H) and 100 MHz (^13^C). ^1^H and ^13^C chemical shifts were referenced internally to solvent shift (acetone-*d*_6_: ^1^H δ 2.05, ^13^C δ 29.8). Chemical shifts (δ are quoted in ppm, the downfield direction being defined as positive. Uncertainties in chemical shifts are typically ±0.01 ppm for ^1^H and ±0.05 for ^13^C. Coupling constants are typically ±0.1 Hz for ^1^H-^1^H and ±0.5 Hz for ^1^H-^13^C couplings. The following abbreviations are used for convenience in reporting the multiplicity of NMR resonances: s, singlet; d, doublet; t, triplet; m, multiplet. All NMR data was acquired and processed using standard Bruker software (Topspin).

### Photophysical measurement

Emission spectra and lifetime measurements for complexes were performed as previously reported method^64^.

### Transmission electron microscopy (TEM) imaging

8 μL of diluted sample solution was applied to a carbon-coated copper grid (T200H-Cu Electron Microscopy Sciences, Washington, USA), dried, and was then negatively stained with 2% uranyl acetate. The stained sample was then allowed to be dried. Transmission electron micrographs were recorded using a Technai G2 Transmission Electron Microscope (FEI, USA) with an acceleration voltage of 200 kV.

### Total internal reflection fluorescence microscopy (TIRFM) imaging

This experimental system and operation was as previously described^52^. After the incubation period, the organic dye ThT (the mole ratio of Aβ_1–40_ and ThT is 1:2) was added to label the Aβ_1–40_ fibrils. Then, a 445 nm diode laser (50 mW, LQC445-40E, Newport, USA) was used for the excitation of the ThT-labeled Aβ_1-40_. Images were obtained by using the WinSpec/32 software (Princeton Instruments, Version 2.5.22.0, Downingtown, PA).

### Preparation of stock solution of tested Ir(III) complexes

The stock solution of all the tested Ir(III) complexes are 10 mM in DMSO.

### Emission measurement in buffered solution

Ir(III) complexes (2 μM) and different concentrations of Aβ_1–40_ monomer/fibril were added into phosphate buffer (50 mM Na_2_HPO_4_, pH 7.4). The mixtures were allowed to equilibrate at room temperature for 3 min without degassing. Emission spectra were recorded on PTI TimeMaster C720 Spectrometer.

### Preparation of Aβ_1–40_ fibrils for seeding

The incubation of Aβ_1–40_ fibrils and seeds used the previously reported method^52^.

### ThT and Ir(III) complexes luminescence binding assays

50 μM of ThT/complex solution (1%) was incubated with 25 μM Aβ_1–40_ monomer solution in phosphate buffer at 37 °C. The luminescence intensity at different time points was measured on a PTI TimeMaster C720 Spectrometer.

### Mass spectrometry experiments

A solution of containing **13** or **14** (25 μM, final concentration) (final DMSO concentration ≤1%) and Aβ_1–40_ peptide monomers (50 μM, final concentration) were mixed in 1 mM ammonium acetate (pH 7.6), and injected into the ESI-TOF-MS at a rate of 3 L/min. ESI-TOF-MS experiments were conducted in the negative-ion mode with a Bruker MicrOTOFQ mass spectrometer. The capillary voltage was set at +3500 V, and the dry N_2_ gas flow was 4.0 L/min at 100 °C. Data were analyzed by the software Bruker Daltonics Data Analysis.

### Cell viability analysis

The cell viability analysis is referenced from a reported method^52^. Specifically, SH-SY5Y cells or primary cortical cells were plated into 96-well plates at a density of 5 × 10^4^ cells/well in cell culture medium. At the next day, the culture medium was replaced with the same medium with 0.2% serum and containing 10 μM **12** and **14** together with monomer, mixture of monomer and seed, and seeding fibril respectively in 1-to-0.2, 1-to-1 or 1-to-5 ratio (final concentration of DMSO ≤ 1.6%). Cells were incubated at 37 °C for 2 and 24 h, respectively. The medium of each well was replaced with 3-(4,5-dimethylthiazol-2-yl)-2,5-diphenyltetrazolium bromide (MTT) solution (5 mg/mL) and further incubated for 3 h at 37 °C. The MTT solution was aspirated off and then 100 μL of DMSO was added to each well to dissolve the formazan crystals. The plates were agitated on a plate shaker for 15 min and the absorbance was measured at 570 nm using a SpectraMax M5 microplate reader (Molecular Devices). Wells without cells were used as blanks and were subtracted as background from each sample. Results were expressed as a percentage of control.

## Additional Information

**How to cite this article**: Lu, L. *et al.* Inhibition of Beta-Amyloid Fibrillation by Luminescent Iridium(III) Complex Probes. *Sci. Rep.* **5**, 14619; doi: 10.1038/srep14619 (2015).

## Supplementary Material

Supporting information
