# Supplementary material for: Inhibition of Beta-Amyloid Fibrillation by Luminescent Iridium(III) Complex Probes
Source: Sci Rep. 2015 Sep 30;5:14619. doi: 10.1038/srep14619 (PMC4588514; doi:10.1038/srep14619)
Supplement: Supporting information [file srep14619-s1.pdf]

Supporting Information

Inhibition of Beta-Amyloid Fibrillation by Luminescent Iridium(III) Complex Probes

Lihua Lu[1]§, Hai-Jing Zhong[2]§, Modi Wang[1], See-Lok Ho[1], Hung-Wing Li*[,1], Chung-Hang Leung*[,2], Dik-Lung Ma*[,1]

[1]Department of Chemistry, Hong Kong Baptist University, Kowloon Tong, Hong Kong, China. E-mail: edmondma@hkbu.edu.hk, hwli@hkbu.edu.hk

[2]State Key Laboratory of Quality Research in Chinese Medicine, Institute of Chinese Medical Sciences, University of Macau, Macao, China. E-mail: duncanleung@umac.mo

§ These authors contribute equally to this work.

Experimental methods

Photophysical measurement. Emission spectra and lifetime measurements for complexes were performed on a PTI TimeMaster C720 Spectrometer (Nitrogen laser: pulse output 337 nm) fitted with a 380 nm filter. Error limits were estimated: λ (±1 nm); τ (±10%); φ (±10%). All solvents used for the lifetime measurements were degassed using three cycles of freeze-vac-thaw.

Luminescence quantum yields were determined using the method of Demas and Crosby[1] $[Ru(bpy)_3][PF_6]_2$ in degassed acetonitrile as a standard reference solution (Φ_r = 0.062) and calculated according to the following equation:

$$\Phi_s = \Phi_r(B_r/B_s)(n_s/n_r)^2(D_s/D_r)$$

where the subscripts s and r refer to sample and reference standard solution respectively, n is the refractive index of the solvents, D is the integrated intensity, and Φ is the luminescence quantum yield. The quantity B was calculated by $B = 1 - 10^{-AL}$, where A is the absorbance at the excitation wavelength and L is the optical path length.

Cytotoxicity test (MTT (3-(4,5-dimethylthiazol-2-yl)-2,5-tetrazolium bromide) assay). Neuroblastoma cells (SH-SY5Y) were seeded in a 96-well flat-bottomed microplate at 8000 cells/well in 100 μL of minimal essential medium (MEM-Eagle, Sigma) containing 10% fetal bovine serum (Invitrogen) and 1% antibiotic and

antimycotic Solution (Sigma). **14** were dissolved in DMSO and mixed with the growth medium (final DMSO concentration ≤ 1.6%). Serial dilutions of each complex were added to each well. The microplate was incubated at 37 °C, 5% CO_2, 95% air in a humidified incubator for 2/6/24 h. After incubation, 10 μL MTT reagent (5 mg/mL) was added to each well. The microplate was re-incubated at 37 °C in 5% CO_2 for 4 h. Solubilization solution (10% SDS, 0.01 M HCl) (100 μL) was added to each well. The microplate was further incubated for 18 h. The absorbance at 570 nm was measured using a microplate reader. The IC_{50} values of **14** (concentration required to reduce the absorbance by 50% compared to the controls) were determined by the dose-dependence of surviving cells after exposure to the **14** for 2/6/24 h.

Synthesis. The following complexes were prepared according to (modified) literature methods. All complexes are characterized by ^{1}H-NMR, ^{13}C-NMR, high resolution mass spectrometry (HRMS) and elemental analysis.

Complex **1**. [Ir(ppy)$_2$(bpy)]PF$_6$ (where ppy = 2-phenylpyridine, bpy = 2,2'-bipyridine) Reported[2]

Complex **2**. [Ir(ppy)$_2$(biq)]PF$_6$ (where biq = 2,2'-biquinoline) Reported[3]

Complex **3**. [Ir(ppy)$_2$(dmdpphen)]PF$_6$ (where dmdpphen = 2,9-dimethyl-4,7-diphenyl-1,10-phenanthroline) Reported[4]

Complex **4**. [Ir(ppy)$_2$(dbbpy)]PF$_6$ (where dbbpy = 4,4'-diphenyl-2,2'-bipyridine) Reported[5]

Complex **5**. [Ir(phq)$_2$(dpbpy)]PF$_6$ (where phq = 2-phenylquinoline) Reported[5]

Complex **6**. [Ir(phq)$_2$(4,7-dpphen)]PF$_6$ (where dpphen = 4,7-diphenyl-1,10-phenanthroline) Reported[4]

Complex **7**. [Ir(bzq)$_2$(chlorophen)]PF$_6$ (where bzq = benzoquinone, chlorophen = 5-chloro-1,10-phenanthroline) Reported[6]

Complex **8**. [Ir(bzq)$_2$(phen)]PF$_6$ (where phen = 1,10-phenanthroline) Reported[7]

Complex **9**. [Ir(bzq)$_2$(2,9-dpphen)]PF$_6$ (where 2,9-dpphen = 2,9-diphenyl-1,10-phenanthroline) Reported[6]

Complex **10**. $[Ir(bzq)_2(aminephen)]PF_6$ (where aminephen = 1,10-phenanthrolin-5-amine) Reported[8]

Complex **11**. $[Ir(bzq)_2(dnbpy)]PF_6$ (where dnbpy = 4,4'-dinonyl-2,2'-bipyridine) Reported[7]

Complex **12**. $[Ir(ppy)_2(phenyl\text{-}imidazo\text{-}phen)]PF_6$ (where phenyl-imidazo-phen = 2-phenyl-1*H*-imidazo[4,5-*f*][1,10]phenanthroline) Reported[9]

Complex **13**.
$[Ir(ppy)_2(phenol\text{-}imidazo\text{-}phen)]PF_6$ (where phenol-imidazo-phen = 4-(1*H*-imidazo[4,5-*f*][1,10]phenanthrolin-2-yl)phenol) Yield: 61%. ^{1}H NMR (400 MHz, Acetone-d_6) δ 9.31 (d, *J* = 7.6 Hz, 1H), 9.23 (d, *J* = 8.0 Hz, 1H), 8.99 (s, 1H), 8.34 (d, *J* = 8.0 Hz, 2H), 8.24 (d, *J* = 8.0 Hz, 4H), 8.07 (s, 1H), 8.00 (s, 1H) 7.95-7.88 (m, 4H), 7.73 (d, *J* = 4.0 Hz, 2H), 7.10-7.04 (m, 4H), 7.00-6.96 (m, 4H), 6.47 (d, *J* = 8.0 Hz, 2H); ^{13}C NMR (100 MHz, Acetone-d_6) δ 168.7, 160.6, 154.5, 151.4, 150.4, 149.6, 145.4, 145.2, 139.4, 133.0, 132.6, 131.2, 129.4, 127.4, 125.8, 124.4, 123.4, 121.9, 120.7, 116.8. HRMS: Calcd. for $C_{41}H_{28}IrON_6$ $[M–PF_6]^+$: 813.1954 Found: 813.1867 Anal. ($C_{41}H_{28}IrON_6OIrPF_6 + 2H_2O$) C, H, N: calcd. 49.55, 3.25, 8.46; found 49.31, 3.13, 8.62.

Complex **14**. $[Ir(bzq)_2(phenol\text{-}imidazo\text{-}phen)]PF_6$ Yield: 52%. ^{1}H NMR (400 MHz, Acetone-d_6) δ 9.22 (d, *J* = 7.6 Hz, 1H), 9.09 (s, 1H), 9.01 (s, 1H), 8.51 (d, *J* = 8.0 Hz, 2H), 8.34 (s, 2H), 8.21-8.18 (m, 4H), 8.01 (d, *J* =8.0 Hz, 4H), 7.88 (d, *J* =8.0 Hz, 2H), 7.60 (d, *J* =8.0 Hz, 2H), 7.46 (s, 2H), 7.24 (t, *J* = 4.0 Hz, 2H), 7.08 (d, *J* =8.0 Hz, 2H), 6.48 (d, *J* =8.0 Hz, 2H); ^{13}C NMR (100 MHz, Acetone-d_6) δ 159.6, 156.4, 153.3, 148.8, 147.2, 140.3, 137.5, 133.7, 129.7, 129.5, 128.5, 128.4, 128.1, 126.8, 126.7, 124.2, 122.7, 120.4, 120.3, 115.9, 115.8. HRMS: Calcd. for $C_{45}H_{28}OIrN_6[M–PF_6]^+$: 861.1954 Found: 861.1963 Anal. ($C_{45}H_{28}ON_6IrPF_6 + 4H_2O$): calcd. C 50.99, H 3.23, N 7.93; found 50.99, 3.18, 8.22.

Table S1 Photophysical properties of **1**–**14**.

Complex	Quantum yield	λ_{em}/ nm	Lifetime/ μs	UV/vis absorption λ_{abs} / nm (ε/ $dm^3mol^{-1}cm^{-1}$)
**1**	0.1278	583.5	4.325	265 (1.13×10^5)
**2**	0.20	656	0.75[3]	275 (1.9×10^5), 355 (6.8×10^4), 376 (7.2×10^4)
**3**	0.2152	568	3.692[4]	255 (3.8×10^4), 326 (1.6×10^4)
**4**	0.1674	591[5]	4.367	205 (9.3×10^4), 255 (7.9×10^4), 305 (3.5×10^4)
**5**	0.2512	560	4.586	278 (1.34×10^5), 355 (1.92×10^4), 454 (4.0×10^3)
**6**	0.058	565	2.39[4]	278.5 (4.2×10^4), 332 (1.8×10^4)
**7**	0.054	580	4.492	267(3.8×10^4), 320(1.6×10^4),
**8**	0.2573	569	186[7]	326(2.06×10^4), 417(5.7×10^3)
**9**	0.13	580	4.843	231 (3.9×10^4), 257 (3.15×10^4), 304 (2.3×10^4)
**10**	0.015	567	25.02[8]	215 (1.4×10^5), 252 (8.6×10^4), 278 (6.3×10^4), 396 (1.6×10^4)
**11**	0.04179	566	4.7[7]	330 (6.9×10^3), 419 (1.89×10^3)
**12**	0.24	587	0.704[8]	227 (6.4×10^4), 278 (1.5×10^5), 478 (1.0×10^4)
**13**	0.10146	583	4.515	218 (5.9×10^4), 277 (2.8×10^5), 416 (8.8×10^3)
**14**	0.0806	586	4.502	280 (2.7×10^5), 422 (1.2×10^4)

Figure S1. Luminescence response of **1–12** (2 μM) in the absence or presence of 25 μM Aβ_{1-40} monomer or fibril in phosphate buffer (50 mM Na_2HPO_4, 100 mM NaCl, pH 7.4).

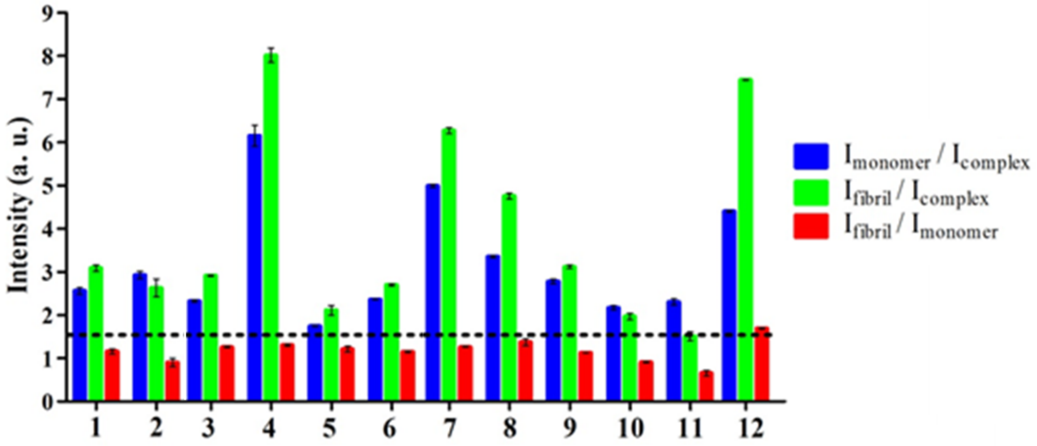

Figure S2. Seed-mediated fibril growth of 25 µM Aβ_{1-40} in the presence of 50 µM of (a) ThT, **12** and (b) ThT, **13**, **14** and (c) ThT, phenol-imidazo-phen, DMSO as measured by luminescence spectroscopy. λ_{Ex} = 310 nm for **12–14**. λ_{Ex} = 435 nm for ThT.

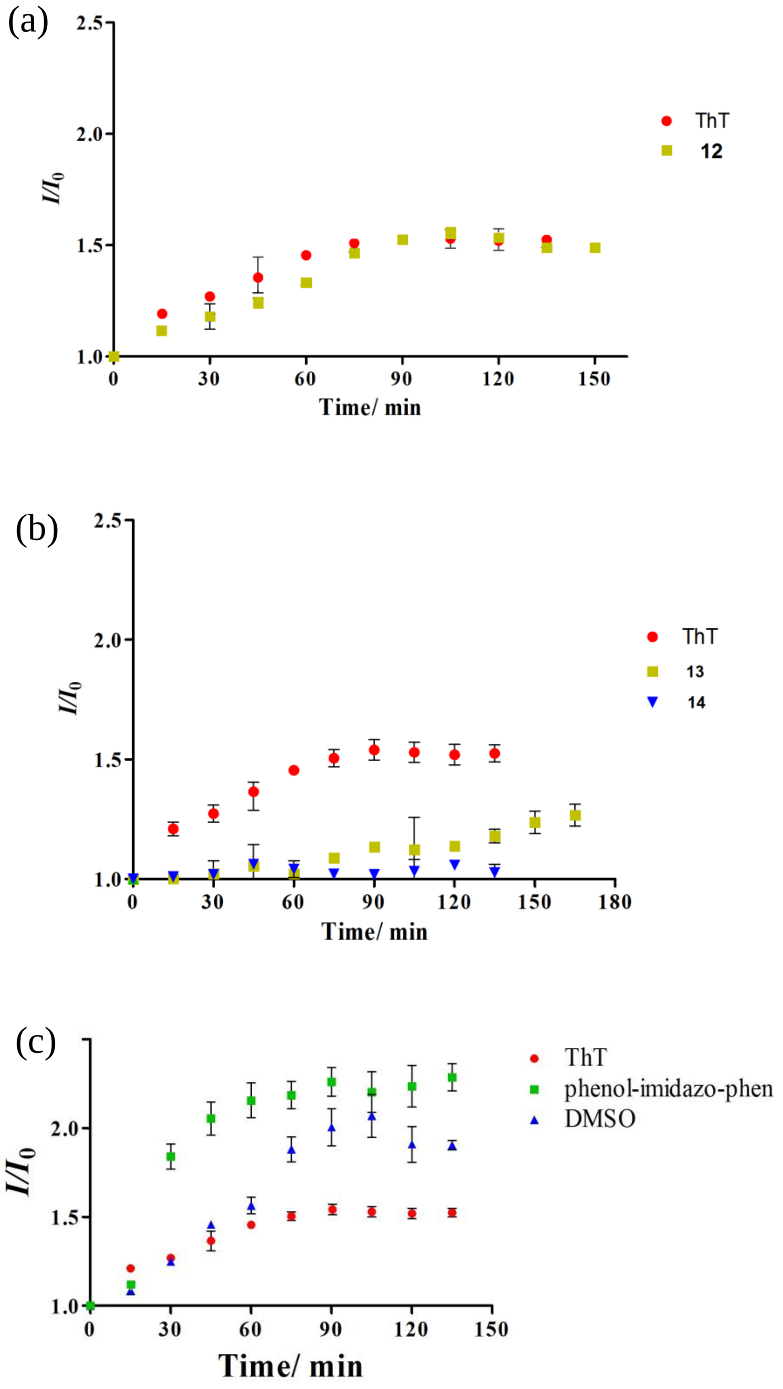

Figure S3. Seed-mediated fibril growth of 25 μM Aβ_{1-40} in the presence of various concentrations of **14** as measured by luminescence spectroscopy.

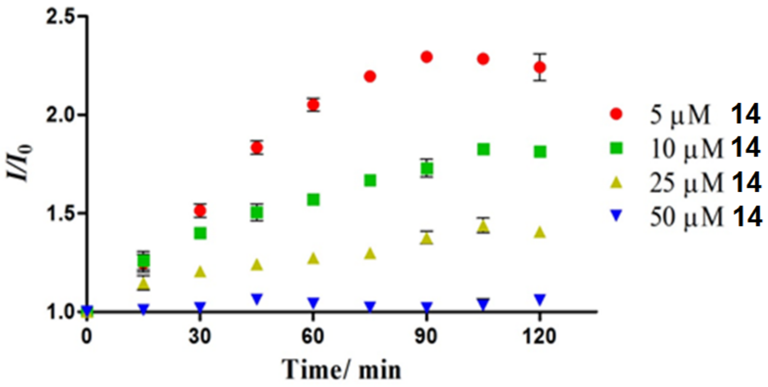

Figure S4. Electrospray ionization time-of-flight mass spectra of 50 μM Aβ_{1-40} peptide incubated in the (a) absence or presence of 25 μM of (b) **13** and (c) **14** in 1 mM ammonium acetate (pH 7.6).

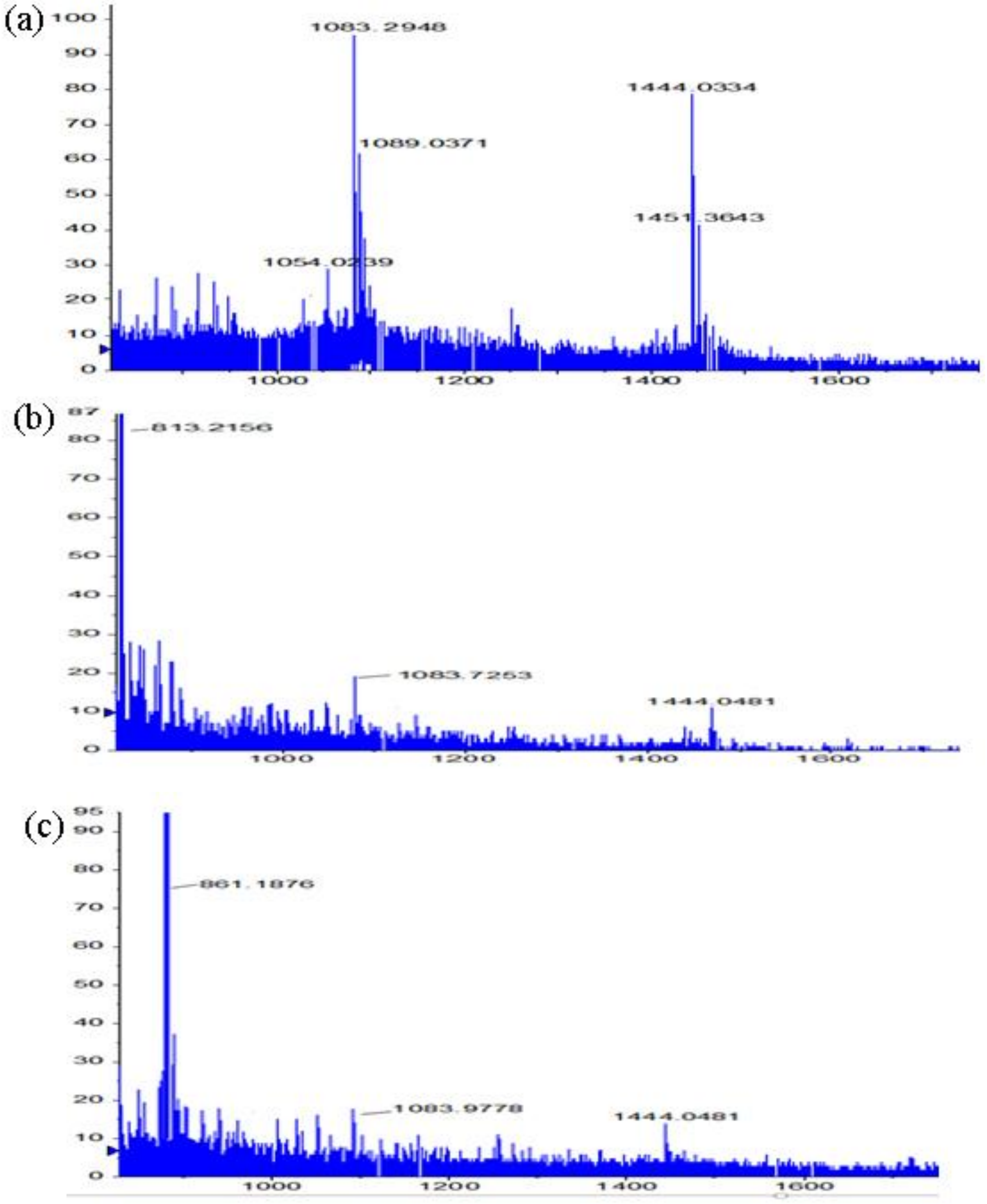

Figure S5. Cytotoxicity studies for **14** with MTT assays. Cytotoxicity of various concentrations of **14** ranged from 1 nM to 100 μM to SH-SY5Y cells incubated for 24 h were measured with MTT assays.

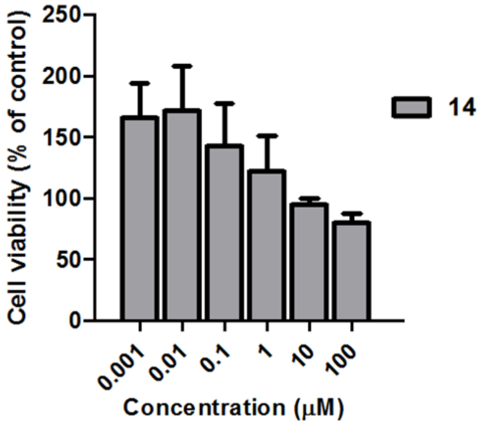

Figure S6. Neuroprotective effect of **12** against A$\beta_{1\text{–}40}$ peptide-induced cytotoxicity towards (a-b) human neuroblastoma SH-SY5Y cells. The histograms show the relative cytotoxicity of various concentrations of A$\beta_{1\text{–}40}$ peptide monomer (M), A$\beta_{1\text{–}40}$ peptide with seeded fibril (MS), and fibrillar A$\beta_{1\text{–}40}$ peptide (F), in the presence of **12**. Various forms of A$\beta_{1\text{–}40}$ peptide were incubated for 2 h at [A$\beta_{1\text{–}40}$]:[**12**] ratios of 0.2:1, 1:1, and 5:1. The relative cytotoxicity was calculated from the cytotoxicity measured for different forms of Aβ peptide with **12** relative to that without, that is, DM/M=(**12**+M)/M; DMS/MS=(**12**+MS)/MS; DF/F=(**12** +F)/F and D=**12**.

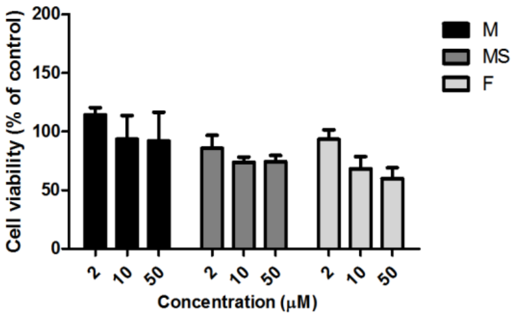

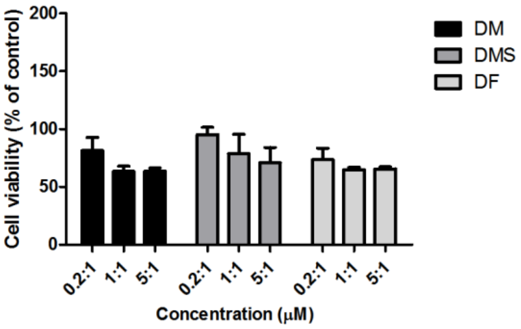

References

1 Crosby, G. A. & Demas, J. N. Measurement of photoluminescence quantum yields. Review. *J. Phys. Chem.* **75**, 991-1024 (1971).

2 Wu, S.-H. *et al.* Dynamics of the Excited States of $[Ir(ppy)_2bpy]^+$ with Triple Phosphorescence. *J. Phys. Chem. A.* **114**, 10339-10344(2010).

3 Su, H.-C. *et al.* Solid-State White Light-Emitting Electrochemical Cells Using Iridium-Based Cationic Transition Metal Complexes. *J. Am. Chem. Soc.* **130**, 3413-3419(2008).

4 Leung, K.-H. *et al.* Label-Free Luminescent Switch-on Detection of Endonuclease IV Activity Using a G-Quadruplex-Selective Iridium(III) Complex. *ACS Appl. Mater. Interfaces* **5**, 12249-12253(2013).

5 Swanick, K. N., Ladouceur, S., Zysman-Colman, E. & Ding, Z. Correlating electronic structures to electrochemiluminescence of cationic Ir complexes. *RSC Adv.* **3**, 19961-19964(2013).

6 Leung, K.-H. *et al.* Detection of base excision repair enzyme activity using a luminescent G-quadruplex selective switch-on probe. *Chem. Commun.* **49**, 5630-5632(2014).

7 Ma, D.-L. *et al.* Antagonizing STAT3 Dimerization with a Rhodium(III) Complex. *Angew. Chem. Int. Ed.*, **53**, 9178-9182(2014).

8 Lo, K. K.-W. *et al.* New Luminescent Cyclometalated Iridium(III) Diimine Complexes as Biological Labeling Reagents. *Inorg. Chem.* **42**, 6886-6897(2003).

9 Zhao, Q. *et al.* Tuning Photophysical and Electrochemical Properties of Cationic Iridium(III) Complex Salts with Imidazolyl Substituents by Proton and Anions. *Organometallics* **26**, 5922-5930(2007).
